# Adoptive transfer of immune cells from glaucomatous mice provokes retinal ganglion cell loss in recipients

**DOI:** 10.1186/s40478-015-0234-y

**Published:** 2015-09-15

**Authors:** Oliver W. Gramlich, Qiong J. Ding, Wei Zhu, Amy Cook, Michael G. Anderson, Markus H. Kuehn

**Affiliations:** Department of Ophthalmology and Visual Sciences, The University of Iowa, Iowa City, 52242 IA USA; Center for the Prevention and Treatment of Visual Loss, Iowa City VA Health Care System, 3135C MERF, 375 Newton Road, Iowa City, IA 52242 USA; Department of Molecular Physiology and Biophysics, The University of Iowa, Iowa City, 52242 IA USA

## Abstract

**Introduction:**

Several studies have indicated that autoimmune and neuroinflammatory processes contribute to the neurodegeneration of retinal ganglion cells in human glaucoma patients and in animal models. To test the involvement of cellular immune processes in the pathophysiology of retinal ganglion cell degeneration *in vivo*, we carried out adoptive transfer experiments from two independent genetic mouse models of glaucoma into normal recipient mice.

**Results:**

Our findings indicate that transfer results in a progressive loss of retinal ganglion cells and their axons despite normal intraocular pressure in recipient mice. Signs of pan-retinal inflammation were not detected. Similar findings were obtained following transfer of isolated T-lymphocytes, but not after transfer of splenocytes from immune deficient glaucomatous mice. Transferred lymphocytes were detected integrated in the spleen and in the retinal ganglion cell layer of recipient animals, albeit at very low frequencies. Furthermore, we observed cell-cell interaction between transferred T-cells and recipient microglia along with focal microglial activation in recipient eyes.

**Conclusion:**

This study demonstrates that the pathophysiology of glaucomatous degeneration in the tested animal models includes T-cell mediated events that are capable of causing loss of healthy retinal ganglion cells.

**Electronic supplementary material:**

The online version of this article (doi:10.1186/s40478-015-0234-y) contains supplementary material, which is available to authorized users.

## Introduction

Glaucoma is a common progressive optic neuropathy and the second leading cause of irreversible blindness in the world. Approximately 60 million people in the world suffer from the disease, and of these, 7 million are blind [44]. Elevated intraocular pressure (IOP) is a major risk factor for the development of glaucoma, but the sensitivity to IOP elevation varies between individuals and a number of pressure-independent factors clearly affect the progression of retinal ganglion cell (RGC) loss in glaucoma [6, 34]. There is mounting evidence that glaucomatous degeneration of the human retina involves immune mediated processes such as the establishment of a pro-inflammatory environment in the eye including activation of the complement cascade [33, 50], microglial activation and expression of MHCII molecules [17, 51, 61, 63], and increased synthesis of pro-inflammatory cytokines such as TNF and nitric oxide [40, 53]. Several studies have indicated that the development of glaucoma is correlated with altered autoantibody patterns in patients [4, 55], suggesting the involvement of a systemic immune response in the disease.

Through the study of animal models it has also become apparent that induction of glaucoma in one eye induces an effect in the naïve contralateral eye. Activation of retinal micro- and macroglia commonly occurs and a mild loss of RGC has been observed in the unmanipulated second eye of mice and rats [15, 30, 47]. Infiltration of monocytes into the optic nerve has been reported in some animal models [22] and it is conceivable that glaucomatous RGC loss induced by elevated IOP initiates a systemic immune response. It has been shown that experimental induction of high IOP levels provokes alteration of the autoantibody pattern [26]. During the last decade, several epitopes for which altered autoantibody reactions have been demonstrated in glaucoma patients were identified. Immunization with those proteins such as heat shock protein 60 and 27 or other retinal/optic nerve head related antigens can induce slow progressive RGC loss in rodent models [24, 57]. Those studies have demonstrated that experimental induction of autoimmunity can lead to RGC loss and appears to involve T-cell mediated Fas/FasL signaling and/or TNF activation [53, 57].

In order to demonstrate that a causal relationship exists between a primed immune system in glaucoma and subsequent, IOP independent, RGC degeneration, we set out to determine if transferred immune cells, derived from glaucomatous animals, are capable of provoking RGC damage in recipients. We utilized mice from two hereditary glaucoma models (B6.*Sh3pxd2b*^*nee*^ and B6SJL;Tg-*MYOC*^*Y437H*^ mice) as splenocyte donors [36, 64]. Both strains are characterized by elevated IOP and progressive RGC loss, but the causative genetic defect is distinct, thereby minimizing the probability that the effect observed in recipient mice is specific for one model system and a direct result of the donor’s mutation. Our data demonstrate that splenocytes from both donor strains cause progressive loss of RGC in normal recipient mice and that T- lymphocytes likely instrumental in this process. These findings provide a causal link between previous studies demonstrating immune activation in glaucoma [4, 17, 18, 52, 54, 55] and those indicating that experimental induction of autoimmunity can result in RGC loss [24, 25, 27, 28, 56–58].

## Materials and methods

### Animals

All animal experimentation was carried out in accordance to the ARVO Statement for the Use of Animals in Ophthalmology and Vision Research and approved by the Institutional Animal Care and Use committee of the University of Iowa. Mice were housed in a 12/12 h day/night cycle and fed *ad libitum*.

Mice carrying a mutation in the gene encoding the podosomal adaptor protein SH3 and PX domains 2B (*Sh3pxd2b*^*nee*^, referred herein as *nee*) are characterized by anterior segment dysgenesis causing elevated IOP, followed by an early-onset, high-penetrance glaucoma phenotype [36]. C57BL/6 mice containing the *nee* mutation were generated by 10 generations of successive backcrosses transferring the mutation from the originating B10.A-*H2*^*h4*^*/(4R)SgDvEg*J background to B6.*Sh3pxd2b*^*nee*^ mice. Subsequently, *nee* mice were intercrossed with B6.129S7*-Rag1*^*tm1Mom*^*/*J *(*referred herein as *Rag1*^*−*^*)* or B6. Cg-Tg(CAG-DsRed*MST)1Nagy/J mice (The Jackson Laboratory) to create immunodeficient *nee/Rag1*^*−*^ mice and homozygote *nee* mice which constitutively expressing the red fluorescent protein DsRed (*nee*/DsRed*).*

Transgenic B6SJL;Tg-*MYOC*^*Y437H*^ (referred herein as *MYOC*) mice were created by crossing C57BL/6 J mice harboring the transgene and SJL mice. Mutations in the myocilin gene are the most common genetic cause of glaucoma in humans [14]. Transgenic *MYOC* mice express a pathogenic variant of human myocilin, which leads to trabecular meshwork dysfunction resulting in the development of moderately elevated IOP and progressive RGC and optic nerve axon loss [64]. Only F1 animals were used for these studies.

IOP was monitored in isoflurane sedated mice using a TonoLab rebound tonometer (Icare, Colonial Medical Supply, Franconia, NH) as previously described [32]. All mice were tested and found to be free of the *rd8* allele, which causes spontaneous retinal degeneration [37].

### Adoptive transfer experiments

For splenocyte transfers, seven-month-old C57BL6/J mice (abbreviated throughout as B6, The Jackson Laboratory, Bar Harbor, ME), two-month-old *nee* mice or *nee/Rag1*^*−*^ mice and twelve-month-old *MYOC* mice were used. Immediately after euthanasia spleens were excised and tissue was mashed gently through a 40 μm pore size nylon cell strainer into a PBS filled petri dish (Greiner Bio One, Monroe, NC). The cell strainer was rinsed with cold 0.1 % BSA/PBS (both Sigma Aldrich, St. Louis, MO) and the splenocyte suspensions were centrifuged at 1500 rpm for 5 min. The pellets were resuspended in 2 ml DMEM buffer (Gibco, Life Technologies, Grand Island, NY) and supernatants were discarded. Cell concentrations were determined using a hemocytometer and adjusted to 10×10^6^ cells/ml. 5×10^6^ splenocytes were injected into recipients via the tail vein. Depending on the background of donor animals, recipient mice were either on a C57BL/6 J or B6:SJL background (non-transgenic F1 littermates of the crosses described above).

T- and B-cell isolation was carried out using splenocytes harvested as described above. Cell pellets containing splenocytes were resuspended in 1 ml cold 0.1 % BSA/PBS after centrifugation and diluted to a concentration of 2×10^6^ cells in1 ml 0.1 % BSA/PBS. FITC anti-CD3 and PE anti-CD19 antibodies and their matching negative control antibodies (BD Biosciences, San Jose, CA) were used for labeling prior to flow cytometry according to the manufacturer’s protocol. Sorting of the splenic CD19 and CD3 lymphocyte fractions was carried out using the FACSAria II system (BD) at the University of Iowa FACS Facility. Following several washing steps in PBS and DMEM buffer 1.5×10^6^ CD19^+^ B-lymphocytes or 1×10^6^ CD3^+^ T-lymphocytes, respectively, were injected into the tail veins of B6 recipients in a volume of 0.5 ml.

An age-matched group of naïve B6 mice (*N* = 7) without transfer were included as additional controls in this experiments.

DsRed positive lymphocytes were obtained from splenocytes harvested from *nee*/DsRed or B6/DsRed mice and processed for FACS sorting as described above, expect that FITC anti-CD3 and BrilliantViolet421anti-CD19 antibodies (both BD) were used for labeling. Again, 1.5×10^6^ DsRed/CD19^+^ or 1×10^6^ DsRed/CD3^+^ lymphocytes were adoptively transferred.

### Optical coherence tomography

OCT imaging was carried out in ketamine/xylazin anesthetized naïve B6 control mice and *nee* splenocyte recipients 6, 12, 18, 24, 42 and 72 days after adoptive transfer using a Bioptigen Envisu OCT (Bioptigen, Morrisville, NC) as described previously [46]. Briefly, OCT was set up with an A-scan by B-scan rate of 1000 and 100 B-scans in a rectangular volume scan with a length of 1.4 mm at a width of 1.4 mm at 0°. The gridded rectangle was adjusted with the papilla as center point.

### Quantification of RGC and axon loss

As reported previously [11], retinas were fixed for 2 h in 4 % paraformaldehyde, dissected, and whole mounted. Retinas were incubated overnight with a rabbit-anti γ-synuclein primary antibody (Abnova, Walnut, CA), followed by secondary antibody incubation with an Alexa Fluor 488 donkey anti-rabbit (Invitrogen, Life Technologies). From each retina a Z-series was taken from six pre-determined mid-peripheral locations using a Nikon Eclipse i80 confocal microscope (Nikon Instruments Inc, Melville, NY) at 600× magnification. Images were stacked, and γ-synuclein^+^ RGC were counted using the cell counter plugin in ImageJ software by an independent observer masked to the animals’ status. This approach offers rapid identification of γ -synuclein positive RGC, although it is conceivable that some stressed RGC down regulate expression of this protein which could exclude those cells from analysis.

The distal parts of optic nerves were fixed in ½ Karnovsiky’s fixative, osmicated, and embedded in Eponate resin. One μm thick sections from each optic nerve were cut with a diamond knife on a Leica EM UC7 ultramicrotome (Leica Microsystems Inc, Buffalo Grove, IL) and stained with 1 % paraphenylenediamine (PPD, Sigma). Photomicrographs were taken at 100× magnification on an Olympus BX41 microscope (Olympus, Center Valley, PA) and assembled. Each optic nerve was independently examined by three investigators masked to the animals’ status and assigned a damage grade based upon the number of damaged axons, the overall organization of the optic nerve, and the frequency of gliotic changes [11, 19]. Grades were defined as: 1 = healthy, 2 = mild damage, no gliosis, 3 = frequent PPD stained axons and mild gliosis, 4 = severe damage with many PPD stained axons and gliosis, 5 = severe damage and large gliotic areas (Additional file 1: Figure S1).

### Immunostaining and Histopathology

All tissues used for immunohistochemistry for histopathology were immersion fixed in 4 % paraformaldehyde. Tissue used for sections was embedded in OCT media. 7 μm sections were obtained and either stained with hematoxylin and eosin or processed for immunohistochemistry. Primary antibodies used include rabbit anti-Iba1 (Wako Chem, Osaka, Japan), CD3 (Rabbit anti-mouse CD3, Abcam, Cambridge, MA) and CD19 (rat anti-mouse CD19, Abcam). Secondary antibodies used were either goat anti-rabbit Cy3 or goat anti-rat Alexa 488 diluted 1:300 (both life technologies). It was also necessary to enhance the endogenous signal of DsRed^+^ lymphocytes through immunohistochemical approaches. Sections were incubated with the primary antibody mouse-anti DsRed (St. Cruz Biotechnologies, Dallas, TX) or rabbit-anti red fluorescent protein (Abcam) and counterstained with DAPI (Sigma, St. Louis, MO) to facilitate orientation.

Retinal whole mounts were preserved in 4 % paraformaldehyde. Retinas where preincubated in 0.3 % Triton X-100/PBS (Sigma) for 4 h and blocked in 0.1 % BSA/0.3 % Triton X-100/PBS for 1 h. Primary antibodies were diluted 1:300 in 0.3 % Triton X-100/PBS and incubated for 18 h at 4 °C. After extensive washing in PBS retinas were incubated with the secondary antibodies (1:200 in PBS) for three hours. Retinal wholemounts were coverslipped and images were taken on an Olympus BX41 microscope.

### Statistics

All data were analyzed in Statistica software (Dell, Round Rock, TX) using Student’s *t*-test for pairwise significance, Tukey’s honest significant difference (HSD) with post hoc tests (with equal and unequal *N*) for multiple comparisons and Kruskal Wallis test for ordinal data. Results are considered statistically significant if *p*-values are less than 0.05. All data are given as mean ± standard deviation (SD).

## Results

### Adoptive transfer of splenocytes derived from mouse models of glaucoma causes RGC loss in recipient animals

In order to test the involvement of an immunologic component in the pathophysiology of RGC degeneration, we carried out a classic adoptive transfer experiment. Splenocytes were obtained from either two-month-old *nee* mice or from healthy B6 animals. In *nee* donor animals (*N* = 10) morphologic abnormalities of the iridocorneal structures leads to a significantly elevated IOP when compared to B6 donor mice (*N* = 10) of similar age *(nee*: 20.2 ± 6.5 vs B6: 11.9 ± 2.5 mmHg; *p* = 0.000008). At this age, retinae of *nee* donors remain structurally intact, but display moderate glaucomatous damage including thinning of the retinal ganglion cell/nerve fiber layer and a pronounced decrease in the RGC density (*nee*: 951 ± 518 RGC/mm^2^, B6: 2394 ± 179 RGC/mm^2^; *p* = 0.000007).

**Fig. 1:**
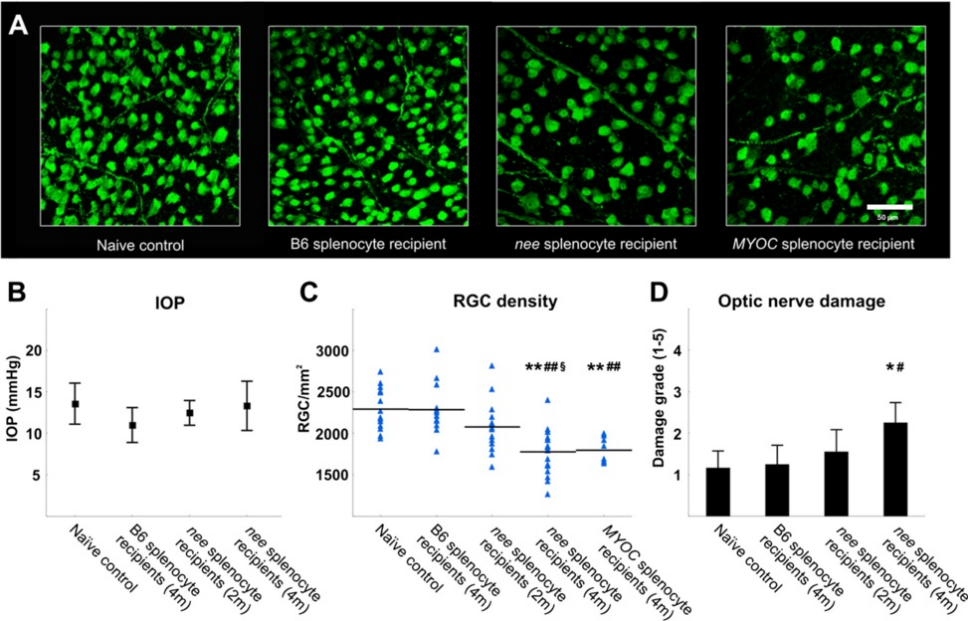
Effects of splenocyte transfer. **a** Immunohistochemical detection of RGC using gamma-synuclein immunohistochemistry in whole mounted mouse retinae. **b** IOP data recorded in naïve control mice, *nee* splenocyte recipient animals two (2 m) and four months (4 m) after transfer, and recipients of healthy B6 splenocyte four months after transfer. IOP in all groups remains within the physiologic range and does not significantly differ between groups (*p* > 0.07). Data are given as mean ± SD. **c** RGC density following adoptive transfer in splenocyte recipients and naïve controls. Splenocyte transfer from healthy B6 donors does not affect RGC density in recipient animals (*N* = 9) four months after transfer. In contrast, animals having received splenocytes from *nee* donor mice suffer a progressive loss of RGC. Two months (2 m, *N* = 7) after transfer RGC density in *nee* splenocyte recipients is slightly reduced, but after four months (4 m, *N* = 10) approximately 23 % of RGC have been lost. RGC damage is also observed in recipient animals (*N* = 4) when splenocytes derived from *MYOC* transgenic mice are transferred. Triangles represent individual RGC data per eye and the horizontal line designates group averages. **Recipient groups vs. naïve control (*N* = 7), *p* < 0.01; ^##^Recipient group vs. B6 recipient animals, *p* < 0.01; ^§^four month recipient group vs. two month recipient group, *p* = 0.02. **d** Average damage scores of optic nerves as determined by PPD staining (1 = healthy optic nerve, 5 = severe damage). Moderate axonal damage is found in optic nerves of *nee* splenocyte recipients four months after transfer. *nee recipient group vs. naïve control, *p* = 0.02; ^#^ *nee* recipient group vs. B6 recipient animals, *p* = 0.02 (Kruskal Wallis test). Data are given as mean ± SD

RGC densities in the recipient animals were determined two (*N* = 14 eyes) and four months (*N* = 20 eyes) after *nee* splenocyte transfer. Data were compared to those derived from mice having received splenocytes from healthy B6 donors (*N* = 14 eyes) as well as to age-matched naïve control mice i.e. no adoptive transfer (*N* = 18 eyes). IOP was monitored in all recipients throughout the study and remained in the physiological range in all groups (average: 12.5 ± 2.7 mmHg, *p* > 0.06, Fig. 1b).

The transfer of splenocytes from *nee* donor mice into B6 mice led to a progressive reduction of RGC numbers in recipient animals (Fig. 1a, c). Two months after transfer a statistically significant difference was not yet observed, although *nee* splenocyte recipients displayed an approx. 10 % decreased RGC density when compared to naïve age-matched controls (2076 ± 316 RGC/mm^2^ vs. 2290 ± 254 RGC/mm^2^, *p* = 0.24). Four months after transfer, *nee* recipient animals demonstrated 23 % RGC loss (1778 ± 267 RGC/mm^2^) when compared to naïve controls (*p* = 0.0001) or to animals having received splenocytes from healthy B6 donors (2284 ± 299 RGC/mm^2^, *p* = 0.0001). At this time-point mean RGC density in all but one *nee* splenocyte recipient was clearly below the average values observed in either age-matched controls or B6 splenocyte recipients (Fig. 1c). In contrast, a decline in RGC density was not observed in the retinas of B6 splenocyte recipients when compared to naïve mice (*p* = 0.99), demonstrating that RGC loss is not initiated by splenocyte transfer itself.

In order to confirm these findings and to rule out that the observed effect is restricted to the *nee* mouse model, we repeated the experiment using splenocytes obtained from *MYOC* mice, a distinct IOP-associated genetic glaucoma mouse model. Adoptive transfer of splenocytes from *MYOC* mice into B6 recipients closely recapitulates the findings obtained using *nee* splenocytes. Four months after transfer retinae in recipient animals display a RGC density of 1798 ± 152 RGC/mm^2^ (*N* = 8 eyes), indicating a loss of approximately 22 % when compared to naïve control animals (*p =* 0.005) or B6 splenocyte recipients (*p =* 0.008, Fig. 1b). Again, increased IOP in recipient mice was not observed.

In concordance with the decline in RGC density, the mean optic nerve damage grade after PPD staining in *nee* splenocytes recipients increased from 1.55 ± 0.5 (*N* = 9 optic nerves) two months after transfer to 2.25 ± 0.5 *(N* = 10 optic nerves) after four months (Fig. 1d). This level of damage is significantly higher than that observed in naive age-matched control animals (1.25 ± 0.5; *N* = 6*, p* = 0.014) or mice having received splenocytes from healthy B6 (1.18 ± 0.4; *N* = 8, *p* = 0.014, Kruskal Wallis test).

### *In vivo* imaging reveals no early pathogenic events in recipients

**Fig. 2:**
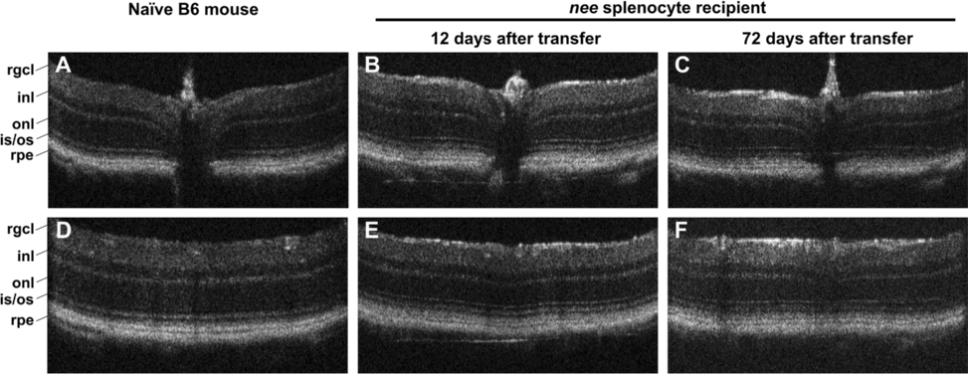
Optical coherence tomography. Retinal OCT imaging demonstrating a normal retinal architecture in the peripapillary (**a**, **b**, **c**) and peripheral regions (**d**, **e**, **f**) in both age-matched B6 naïve mice and a representative *nee* splenocyte recipient 12 and 72 days after transfer. No evidence for acute infiltration or retinal detachments was identified through *in vivo* imaging. A thinning of the retinal ganglion cell layer (rgcl, including the nerve fiber layer) was not yet evident 72 days after transfer, even though at that time a slight loss of RGC is observed using histochemical approaches. However an increased reflectivity (**b**, **c**, **e**, **f**) in the rgcl was noted when nee-transfer animals and naïve mice are compared (inl: inner nuclear layer; onl: outer nuclear layer; is/os: inner and outer photoreceptor cell segments; rpe: retinal pigment epithelium)

Optical coherence tomography (OCT) and funduscopy is routinely used in clinical ophthalmology and in animal research to detect retinal abnormalities *in vivo*. To test whether the damage observed in our model was related to an acute inflammation event, we imaged recipients’ retinae at several time points after splenocyte transfer. Particular attention was directed towards any signs of uveitis, such as retinal detachments, subretinal hemorrhage, or white linear lesions as described elsewhere [10]. Retinal scans and funduscopy were carried out in an independent group of *nee* splenocyte recipients (*N* = 6) 6, 12, 18, 24, 42 and 72 days after transfer and compared to naïve control mice. Retinal folds or signs of cellular infiltrates were not observed in any of the animals after transfer of immune cells, regardless of the time interval after injection. Moreover, recipient animals uniformly display an undisturbed retinal architecture and a normal appearance of the optic nerve head, even at later stages when RGC density has already begun to decline (Fig. 2).

### T-lymphocytes promote RGC loss more vigorously than B-lymphocytes

**Fig. 3:**
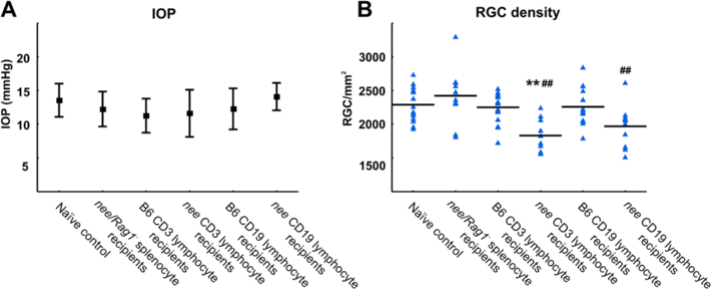
T- and B-cell adoptive transfer. **a** IOP recordings in *nee/Rag1* ^*−*^ splenocyte recipients (n=*,* CD3^+^ and CD19^+^ cell recipient animals. Data indicate that the transfer does not influence IOP and remains similar to IOP values of naïve mice. The mean IOP over all groups remains at 12.6 ± 2.8 mmHg (*p* > 0.5). Data are given as mean ± SD. **b** RGC density in *nee/Rag1* ^*−*^ splenocyte recipients and CD19^+^ or CD3^+^ lymphocyte recipient mice 4 months after transfer. Transfer of *nee* CD3^+^ T-lymphocytes induces a significant loss of RGC in animals (*N* = 5) when compared to recipients of B6 CD3^+^ cells (*N* = 8), to B6 CD19^+^ lymphocyte recipients (*N* = 8), or to a naïve control group (*N* = 7, **p < 0.001). Adoptive transfer of the *nee* CD19^+^ fractions results in a modest, but not statistically significant, reduction of RGC in recipients (*N* = 5, *p* = 0.12) when compared to the corresponding B6 CD19^+^ lymphocyte recipients. Transfer of splenocytes from immunodeficient *nee/Rag1* ^*−*^ donors did not result in loss of RGC in recipients (*N* = 6) when compared to naïve mice and animals having received lymphocytes from B6 donors (*p* < 0.84). Accordingly, RGC density is also significantly lower in *nee* when compared to *nee/Rag1* ^*−*^ animals (^##^ *p* < 0.001). Triangles represent individual RGC data and the horizontal line designates the group average

Following our observation that RGC loss is inducible by adoptive transfer of splenocytes, we sought to determine if the lymphocytes included in these preparations are primarily responsible for the observed RGC loss in recipients and, if so, whether this effect is mediated by B- or T-cells. In order to address this question, we created immunodeficient *nee* mice by crossing them with Rag1^−^ mice [38]. The resultant immunodeficient *nee* (*nee/Rag1*^*−*^) lack mature T-cells or B-cells, but display the glaucomatous phenotype with high IOP (25.2 mmHg) and progressive RGC loss. Splenocytes were obtained from these animals and transferred into B6 recipients (*N* = 6) as described above. In addition, respective CD3 and CD19 positive cell fractions were isolated by flow cytometry from splenocytes of *nee* mice or B6 donors. Recipient B6 mice were injected with either 1×10^6^ CD3^+^ T-lymphocytes, or 1.5×10^6^ CD19^+^ B-lymphocytes from either *nee* (*N* = 5, ea.) or healthy B6 donor animals (*N* = 8, ea.). As noted following splenocyte transfer, adoptive transfer of splenocytes or T- or B-lymphocytes from glaucomatous mice did not increase IOP in the recipient animals. The average IOP of recipient mice was 12.3 ± 2.6 mmHg, similar to the normal population average (13.5 ± 2.5, *p* < 0.5, Fig. 3a).

Data obtained clearly demonstrate that adoptive transfer of splenocytes from T- and B- cell deficient *nee/Rag1*^*−*^ donors does not result in RGC loss in recipients. The RGC density following transfer of *nee/Rag1*^*−*^ splenocytes *was* 2423 ± 383 RGC/mm^2^, very similar to the naïve control group (*N* = 7*,* 2290 ± 254 RGC/mm^2^, *p* = 0.84) and to animals injected with T- or B-lymphocytes from B6 donors (*p* > 0.6). In contrast, mice having received purified CD3^+^ T-lymphocytes from *nee* donors displayed a RGC density of 1831 ± 245 RGC/mm^2^, significantly less than those recipients injected with CD3^+^ T-cells from B6 mice (2251 ± 221 RGC/mm^2^, *p* = 0.005). In contrast, transfer of CD19^+^ B-lymphocytes from *nee* mice only resulted in a moderate reduction of RGC density in recipient animals when compared to mice having received CD19^+^ B-lymphocytes from B6 mice (1968 ± 317 RGC/mm^2^ vs. 2259 ± 266 RGC/mm^2^, respectively, *p* = 0.12, Fig. 3b).

### Transferred lymphocytes are observed at low frequency in recipients’ retinae

To investigate the behavior of transferred lymphocytes in recipient animals, a transgenic allele causing constitutive expression of the red fluorescent reporter protein DsRed was introduced into *nee* mice (*nee*/DsRed). DsRed^+^ lymphocytes were sorted and adoptively transferred as described above. Extravasated DsRed^+^ lymphocytes were detected in whole mounted retinas as early as 16 days after transfer. In each eye, 10–15 DsRed^+^ B-lymphocytes and fewer than 5 DsRed^+^ T-lymphocytes are found in retinal tissue outside blood vessels in *nee*/DsRed lymphocyte recipients. In contrast, DsRed^+^ lymphocytes were not found in the retina of recipients of B6/DsRed T-cells at that time. However, examination of retinal tissue obtained at later stages (20, 28 and 42 days after injection) frequently revealed the presence of a small number (<5) of transferred DsRed^+^ T- and B-lymphocytes regardless of whether they were derived from glaucomatous or healthy donor animals.

**Fig. 4:**
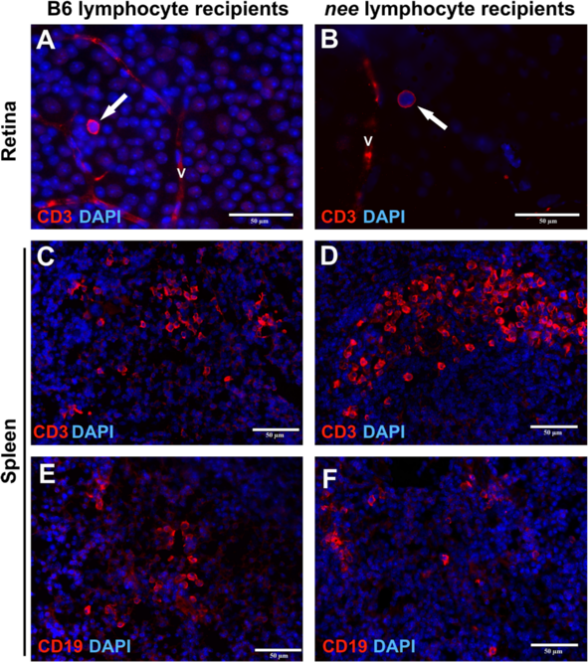
Transferred lymphocytes infiltrate the retina and spleen. **a**, **b** Immunohistochemical detection of DsRed/CD3^+^ T-lymphocytes (red) derived from (**a**) healthy B6 (**b**) *nee* donors in the retina of recipient mice 20 days after transfer. The presence of sporadic extravasated DsRed^+^/CD3^+^ T-lymphocytes located either within the recipients’ RGC layer (shown in a) or epiretinally on the surface of the nerve fiber layer (shown in b) was noted in all recipients. V = retinal vessel. **c**-**f** Infiltration of transferred DsRed^+^ lymphocytes into the spleen of recipient animals 28 days after injection. Integration of immune cells obtained from *nee* donors was noticeably more pronounced than that of those derived from B6 donors. Notation of CD3 or CD19 in the microphotographs refers to the transferred lymphocyte fraction rather than immunohistochemical detection. DAPI was used to label nuclei

Consequently, it appears that transferred T-lymphocytes are able to pass the blood retina barrier and are present in the retinal parenchyma, although at a very low frequency. Furthermore, a dramatic increase in the number of extravasated lymphocytes was not apparent in mice having received adoptive transfers from glaucomatous mice. DsRed^+^ cells could also be detected in blood smears of all recipient mice shortly after transfer and throughout the study. Transferred DsRed^+^ T- and B-lymphocytes were also readily detectable in the spleens of recipient mice where they become integrated into the pulpa in all recipient groups (Fig. 4c-f).

Finally, we examined longitudinal optic nerve sections, as well as brain, liver, and cervical lymph node specimens for the presence of transferred lymphocytes. DsRed^+^ cell were not detected and examination of H&E stained sections did not reveal indications of pathological changes, such as an influx of autologous lymphocytes in retinal (Additional file 2: Figure S2) or other tissues in any of the mice evaluated.

### Microglia become activated early and interact with lymphocytes

**Fig. 5:**
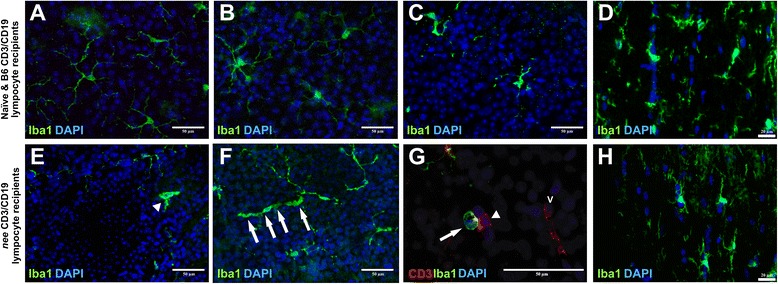
Appearance of ocular microglia in recipients of B6 or *nee* lymphocytes. **a** Iba1 immunostained microglia in the whole mounted retina of an age-matched naïve control mouse depicting quiescent, ramified microglia. Retinal microglia in recipients of (**b**) CD19^+^ and (**c**) CD3^+^ cells donated from B6 mice appear uniformly ramified 28 days after transfer. **d** Horizontal sections of the optic nerve also did not reveal signs of microglial activation. Here the optic nerve of a recipient after adoptive transfer of B6 CD19^+^ cell fraction is shown. Numerous activated microglia are readily identified in recipients of (**e**) CD19^+^ or (**f**) CD3^+^ lymphocytes from *nee* donor mice (bottom row). Both early-stage microglial activation, indicated by hypertrophic somatic areas (arrows in f), and highly activated microglia (arrowhead in e) were observed. **g** Interactions between transferred T-lymphocytes and activated resident microglia are occasionally found. These cells are located outside the retinal vasculature (V) and are accompanied by additional, unidentified, DAPI positive endogenous cells. **h** Microglial activation was not observed in the optic nerve of any recipients. Scale bars in a-c, e-f is 50 μm and in d, h 20 μm

One of the hallmarks of neurodegeneration, including retinal degeneration, is the activation of microglia [35, 41]. As the innate immune cell of the central nervous system, microglia strongly interface with the systemic immune system to cause bidirectional signaling, into and out of the CNS [42]. Hence, we examined if retinal microglia are affected by splenocyte transfer and whether there is evidence of their interaction with transferred cells in the retinas of recipient mice using the microglia marker Iba1 [29]. In the healthy retina microglial cells have a highly ramified phenotype with small soma, thin branches and a fine network of cellular processes. An early stage of microglial activation is indicated by retraction of their branches and hypertrophic somas, whereas fully activated microglia cells are characterized by an amoeboid shape [31]. It was apparent that only retinae of *nee* lymphocyte recipients, but not B6 lymphocyte recipients, contain a small population of hypertrophic Iba1^+^ microglia with an amoeboid shape as well as a few highly activated microglia (Fig. 5). Those microglia that appeared to be activated are frequently observed in close contact to transferred DsRed/CD3^+^ cells from *nee* mice even if quiescent microglia are apparent a short distance away. This interaction between transferred lymphocytes and resident microglia is occasionally accompanied by other, not yet identified, endogenous cells (Fig. 5f). These observations suggest an interaction between CD3^+^ T-lymphocytes and resident microglia.

## Discussion and conclusions

Findings from both clinical and laboratory studies have firmly established that IOP independent mechanisms contribute significantly to the pathophysiology of glaucoma. One important feature that the glaucomatous retina shares with many other neurodegenerative diseases is the accumulation of components of the immune system that occurs subsequent to the onset of the disease [21, 49]. Such neuroinflammatory processes can elicit downstream immune responses that may contribute in important aspects to the pathology as has been described in many neurodegenerative diseases [1, 2].

Immune processes have also been associated with the development of glaucoma, but a causal relationship has not yet been established. Herein, we demonstrate that adoptive transfer of splenocytes or isolated CD3^+^ or CD19^+^ cells obtained from mouse models of glaucoma into normal mice results in progressive RGC loss without IOP elevation in recipient animals. In contrast, transfer of splenocytes from immune deficient glaucomatous mice or healthy B6 donors does not result in RGC loss. While it cannot be ruled out completely that the observed immune response is in some way dependent upon the genetic modification of the models used, the finding that splenocytes from two independent genetic models can mediate the same effect suggests that the establishment of an immune response is a general phenomenon that likely results from elevated IOP and RGC degeneration. Furthermore, the observed damage in recipient mice developed at physiological IOP levels and is consequently independent of elevated IOP.

One important issue of the present study is that the observed RGC loss could have occurred either as part of autoimmune mediated nonspecific pan-retinal degeneration or a rapid early inflammatory event. Neither of these scenarios is consistent with glaucoma pathophysiology and consequently recipient mice were carefully examined to address these concerns. Retinas of recipient mice were monitored noninvasively *in vivo* using optical coherence tomography. Our findings demonstrate that transfer of splenocytes from glaucomatous mice does not result in loss of cells, which is indicated by changes in the thickness of the appropriate layer, outside the ganglion cell/nerve fiber layer. We also did not observe acute episodes of leukocyte infiltration in the retina, as is frequently the case in uveitis [10], or in the optic nerve, as observed in optic neuritis or during experimental autoimmune encephalomyelitis [45]. Spatiotemporal tracking of transferred DsRed labeled lymphocytes revealed integration into the spleen, but invariably rare transferred immune cells have been observed in the ganglion cell layer, regardless of the time interval since injection. Accordingly the cell loss in recipient mice is slow, progressive and RGC specific. As such the observed events are consistent with glaucoma pathology rather than those of classical immune mediated ocular diseases.

The retina is a part of the central nervous system and consequently is an immune privileged tissue [16, 59]. Ocular immune privilege is physiologically maintained by the blood/retina barrier, but microglia also modulates the process by secretion of pro- or anti-inflammatory cytokines. The microglial cytokine profile in turn can be significantly influenced by signaling from lymphocytes [8]. It has recently become appreciated that T-lymphocytes crossing the blood/retina barrier is a normal process which occurs continuously albeit at a low rate even in healthy eyes [9, 43]. We previously demonstrated the presence of lymphocytes in the retinal parenchyma of eyes obtained from human glaucoma patients [17]. Our findings in this study are congruent with these data: a very small number of transferred T- and B-lymphocytes derived from either control or glaucomatous mice are detectable in the ganglion cell layer of recipient mice.

It is intriguing to speculate how adoptive transfer of T- or B-lymphocytes can lead to the observed progressive RGC loss. Our findings clearly ascribe a functional role to CD3^+^ donor cells, but adoptive transfer of CD19^+^ cells also resulted in reduced RGC density in recipient animals, although statistical significance was not reached. Identification of the active lymphocyte cell type is complicated by the slow progression of damage, requiring an extended experimental period that allows ample opportunities for cross-talk between the donated T- or B-cells and the immune system of the recipient. Furthermore, the small number of extravasated lymphocytes in recipient retinas casts some doubt on whether RGC are damaged directly by T-lymphocytes. While it is conceivable that the steady activity of even a small number of effector T-cells could significantly degrade the number of surviving RGC cells over the lengthy observation period, an alternative explanation is also possible. Microglial activation is a common response to retinal injury and also occurs in glaucoma [5, 12]. In this study we observed direct interaction of epiretinal T-cells with activated tissue microglia, similar to our earlier observations in human eye donor tissue [17], and it is possible that the neurodegenerative effect is due to the deleterious activities of microglia that become activated due to T-cell signaling. Microglial activation entails the release of potentially RGC damaging substances such as TNF-α, nitric oxide synthase-2, and Fas-ligand [62] that could compromise the health of additional RGC [48, 57]. In this scenario, the role of T-cells is restricted to immunologic memory and a large number of lymphocytes within the neural retina is not required to cause the establishment of a damaging pro-inflammatory environment. Interestingly, a number of studies have indicated that CNS degenerating diseases can be exacerbated by a pro-inflammatory environment, even if the cause of the inflammation is not directly related to the disease. For example, systemic inflammation accelerates the progression of brain inflammation and frequently precedes relapses in multiple sclerosis patients [39, 42]. It appears that a general pro-inflammatory environment can enhance neuronal damage via the detrimental activities of activated microglia. In this respect it is interesting to note that a recent report indicated a correlation between oral bacterial counts, microglial activation, and vision loss in glaucoma patients [3]. Retinal microglia may also participate in the initial establishment of the autoimmune response. These cells are likely involved in the phagocytosis of damaged RGC and in antigen presentation. Phagocytosed cellular debris is then loaded onto antigen presenting MHC class II molecules, which are expressed at high levels by microglia in the glaucomatous retina [15, 23]. T-lymphocytes may encounter the presented antigens either during normal surveillance or following failure of the retinal vasculature, as in the case of splinter disk hemorrhage, a complication of glaucoma which is significantly associated with disease progression [13]. Primed T-cells then return to the lymphoid organs where they interact with other lymphocytes and stimulate the maturation of effector T-cells, which subsequently return to the eye to degrade additional RGC. Thus the development of an autoimmune response follows initial damage, but establishes an IOP independent mechanism of RGC loss.

The clinical management of glaucoma patients is all too frequently beset by slow and gradual vision loss that continues even at IOP below the population average [7, 20] and there are indications that the T-cell profile in glaucoma patients is distinct from healthy controls [60]. If secondary autoimmune events akin to those described herein occur in glaucoma patients, current treatment modalities are ill equipped to minimize their detrimental effects which could explain continued or recurring episodes of vision loss in the absence of elevated IOP.

## Additional files

Additional file 1: Figure S1. Representative images of PPD stained optic nerve sections used to assess axonal damage. With increasing axonal damage stained axons (arrowhead in B) become more numerous and gliotic areas (arrow in C) increase. (A) Naïve control (Damage grade 1) (B) B6 splenocyte recipient (Damage grade 2) (C) nee splenocyte recipient (Damage grade 3). (TIFF 135 kb) Additional file 2: Figure S2. Representative images of H&E stained retinal cross –sections. Examinations of retinal cross-sections show the typical layered structure without any indications of severe pathological changes. The morphology of the retina in age-matched naïve control animals and in nee splenocyte recipients, four months after transfer, are in accordance with the retinal phenotype examined through OCT. (rgcl, including the nerve fiber layer: inl: inner nuclear layer; onl: outer nuclear layer; is/os: inner and outer photoreceptor cell segments). (TIFF 3627 kb)
