# Supplementary figures and images for: Adoptive transfer of immune cells from glaucomatous mice provokes retinal ganglion cell loss in recipients
Source: Acta Neuropathol Commun. 2015 Sep 15;3:56. doi: 10.1186/s40478-015-0234-y (PMC4591529; doi:10.1186/s40478-015-0234-y)

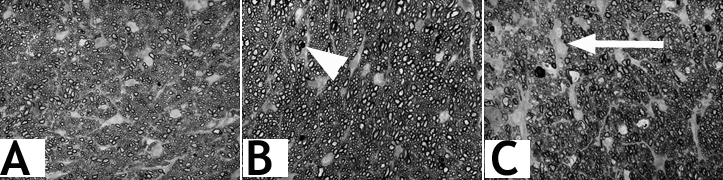

Supplement: Additional file 1: Figure S1. — Representative images of PPD stained optic nerve sections used to assess axonal damage. With increasing axonal damage stained axons (arrowhead in B) become more numerous and gliotic areas (arrow in C) increase. (A) Naïve control (Damage grade 1) (B) B6 splenocyte recipient (Damage grade 2) (C) nee splenocyte recipient (Damage grade 3). (TIFF 135 kb) [file 40478_2015_234_MOESM1_ESM.tif]

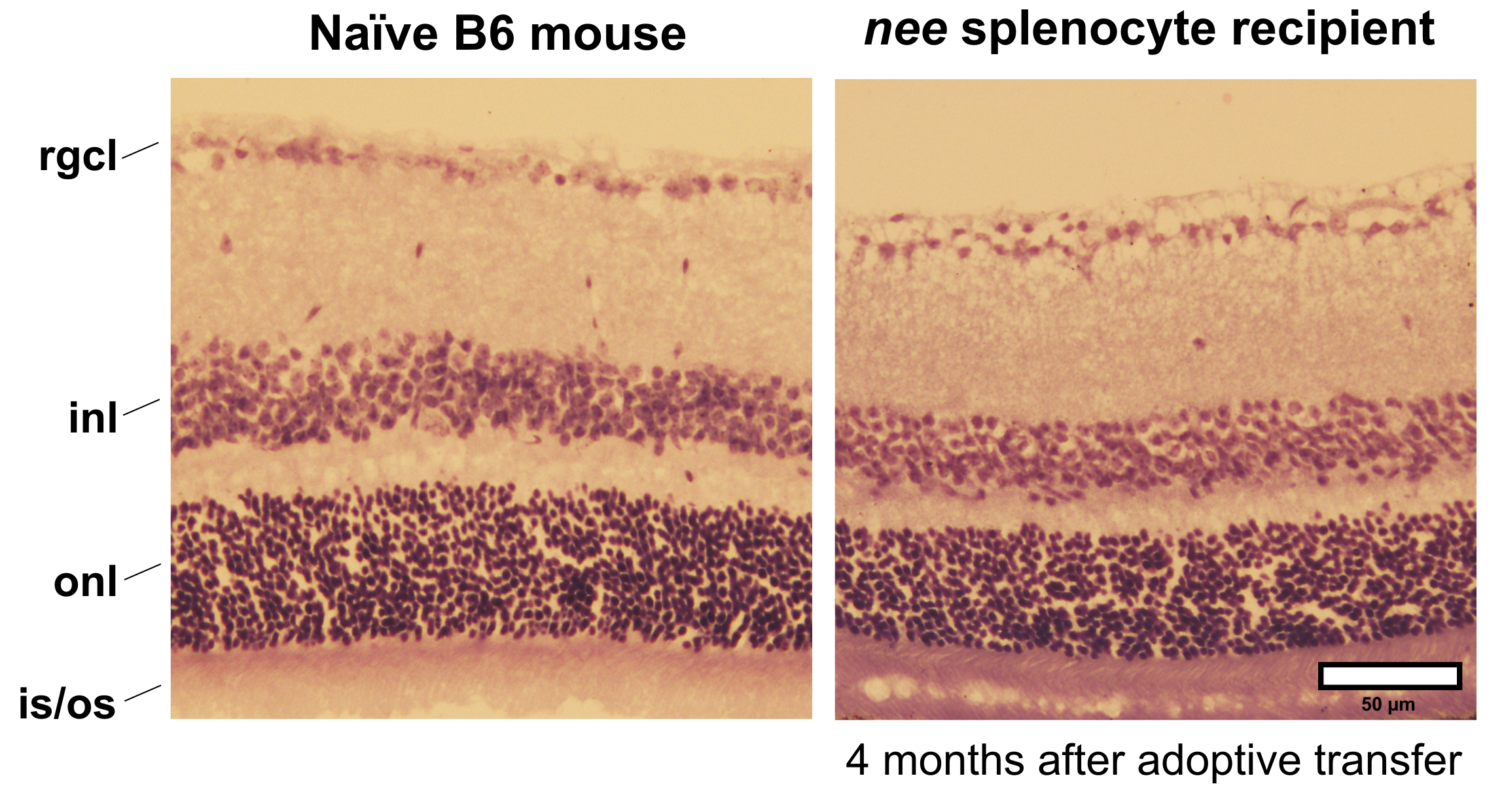

Supplement: Additional file 2: Figure S2. — Representative images of H&E stained retinal cross –sections. Examinations of retinal cross-sections show the typical layered structure without any indications of severe pathological changes. The morphology of the retina in age-matched naïve control animals and in nee splenocyte recipients, four months after transfer, are in accordance with the retinal phenotype examined through OCT. (rgcl, including the nerve fiber layer: inl: inner nuclear layer; onl: outer nuclear layer; is/os: inner and outer photoreceptor cell segments). (TIFF 3627 kb) [file 40478_2015_234_MOESM2_ESM.tif]
